# Interleukin-6 polymorphisms in HCC patients chronically infected with HCV

**DOI:** 10.1186/s13027-020-00285-9

**Published:** 2020-04-01

**Authors:** Faisal Adnan, Najeeb Ullah Khan, Aqib Iqbal, Ijaz Ali, Arnolfo Petruzziello, Rocco Sabatino, Annunziata Guzzo, Giovanna Loquercio, Gerardo Botti, Sanaullah Khan, Muhammad Naeem, Muhammad Ismail Khan

**Affiliations:** 1 Institute of Biotechnology and Genetic Engineering (Health Division), The University of Agriculture, Peshawar, Pakistan; 2 Department of Biosciences, COMSATs University, Islamabad, Pakistan; 3 UOC Clinical Pathology, AORN Sant’Anna e San Sebastiano, Caserta, Italy; 4 Unit of Molecular Biology and Viral Oncology, Istituto Nazionale Tumori, IRCCS Fondazione Pascale, Naples, Italy; 5 SSD Transfusion medicine, Istituto Nazionale Tumori – IRCCS Fondazione “G. Pascale”, Naples, Italy; 6 Hematology-Oncology and stem cell transplantation Unit, IRCCS Fondazione “G. Pascale”, Naples, Italy; 7 Scientific Directorate, Istituto Nazionale Tumori, IRCCS Fondazione “G. Pascale”, Naples, Italy; 8 Department of Zoology, University of Peshawar, Peshawar, KP Pakistan; 9 Department of community medicine, Khyber Medical Collage, Peshawar, Pakistan; 10 Department of Zoology, Islamia College, Peshawar, Pakistan

**Keywords:** HCC, Interleukin-6, SNP, Risk factors

## Abstract

Hepatocellular carcinoma is a primary liver malignancy in which the risk of development is always multifunctional. Interleukin-6 is a proinflammatory and multifunctional cytokine, which plays an important role in the immune response, haematopoiesis and defence against viral infection. We aimed to evaluate the frequency of *Interleukin-6* mutations (rs2069837 and rs17147230) associated with genetic risk of hepatocellular carcinoma in Khyber Pakthunkhwa population. A total of 72 hepatocellular carcinoma cases and 38 controls were included in this study. The genomic DNA was extracted from the peripheral blood cells and *Interleukin-6* genotyping was performed using T-ARMS-PCR technique. Our results show a significant increase risk of developing hepatocellular carcinoma with the mutation within *Interleukin-6* gene with heterozygous G allele (rs2069837) (OR = 10.667, 95%CI = 3.923–29.001, *p* = < 0.0001) and heterozygous T allele (rs17147230) (OR = 75.385, 95%CI = 9.797–580.065, *p* = < 0.0001). However, under recessive gene model the results were insignificant in case of *Interleukin-6* rs2069837 (OR = 0.605, 95%CI = 0.217–1.689, *p* = 0.337), while significant in case of *Interleukin-6* rs17147230 (OR = 0.298, 95%CI = 0.121–0.734, *p* = 0.0085). In conclusion, *Interleukin-6* mutation is associated with hepatocellular carcinoma susceptibility. More related studies with other associated interleukins and their whole gene sequencing will be required.

## Background

Hepatocellular carcinoma (HCC) is the most common primary liver cancer. In Pakistan, HCC represents the fourth most common malignancy in men and the seventh in woman [1]. Worldwide, HCC is considered the third main cause for cancer death, especially in patients with chronic hepatitis B virus (HBV) and hepatitis C virus (HCV) infection [2, 3]. In advanced stages of HBV and HCV diseases the immune response is often insufficient to eradicate the viruses, resulting in chronic liver inflammation through a lifelong host-virus interaction. There is growing evidence that chronic inflammation is involved in the progression of cancer. HCC occurrence varies greatly around the world but HCC is found 3–4 times more frequent in males often than females [4]. This difference in gender may be due to lifestyle-related risk factors for HCC, such as alcohol consumption and smoking. Though, sex hormones and X-linked chromosome hereditary factors may also be important, because males are more vulnerable to HCC than females [5]. It has also been demonstrated that host genetic factors, such as single nucleotide polymorphisms (SNPs), could affect individual susceptibility to HCC [6]. The risk factors which include diabetes, obesity and environmental factors like carcinogens are observed highly variable among the HCC patients, which are mostly associated with the race or ethnic groups and geographic region of the infected individuals. However, majority of these risk factors progress to the development of cirrhosis, which is present in almost 80–90% of patients with HCC [7].

Macrophages and lymphocytes produce interleukins (ILs) and other cytokines that regulate antiviral activity genome-wide. Association of SNPs at various ILs have shown positive influence on chronic HCV leading to HCC development [8]. Various ILs and their respective SNPs have shown positive association with HCV treatment and susceptibility, which leads to cirrhosis, fibrosis and HCC development. Cytokines act in a very complex and composed system in which they initiate or block their own synthesis and additionally, different cytokines and cytokine receptors synthesis [9]. In any case, there is growing evidence concerning the role of hereditary components to imbalance pro-inflammatory and anti-inflammatory cytokine profile that may influence the clinical result and severity of Hepatitis C. The balance of pro-inflammatory and anti-inflammatory cytokines may change the benefits of antiviral treatment, thereby affecting the outcome of disease, for example, the clearance of HCV after severe infection or the development of liver disease [10]. However, there is no straightforward evidence about the association of ILs and their respective SNPs with viral infections within ethnic groups of Khyber Pakthunkhwa (KP) population.

Interleukin-6 (IL-6) is a pro-inflammatory and multifunctional cytokine, which is located at 7p21 chromosome. Hepatic response to infections and systemic inflammation is the key function of IL-6 [11]. Indeed, IL-6 produced by B cells, T cells, macrophages, and fibroblasts have a pleiotropic effect on inflammation, immune response, and haematopoiesis [12]. Polymorphisms in *IL-6* promoter region were appeared to be involved in the pathogenesis of some immune mediated diseases [13]. The association of SNPs within the *IL-6* gene have shown different response to HCC and proliferation with respect to alcoholic, HBV and HCV infections [14]. However, there is still a gap to understand the association of *IL-6* SNPs within ethnic groups, including Pakistani population with HCC progression and proliferation in chronic HBV and HCV infections. Therefore, understanding the HCC development in chronically HCV infected individuals and their associated risk factors would be a great importance in HCC progression. ILs and other genetic factors including microRNAs and mitochondrial DNA association with treatment regimen have great importance nowadays. However, there is no such evidence about the association of *IL-6* SNPs and HCC patients in KP population, which are chronically infected with HCV. In the present study, we will explain the association of *IL-6* SNPs with HCC susceptibility in chronically HCV infected individuals of KP-Pakistan.

## Methods

### Demographic data and blood sampling

Demographic data and blood samples were collected from 80 HCV related HCC and 50 HCV infected patients at Leady Ready Hospital and Hayatabad Medical Complex Peshawar, KP, Pakistan during January to December 2018. Based on our study inclusion/exclusion criteria (HCV leading to HCC), 72 out of 80 HCC patient and 38 out of 50 HCV infected samples as control were selected for further analysis.

### HCV screening and RT-PCR

All the samples were confirmed for HCV using Immuno-Chromatographic Test (ICT) and PCR. Briefly, serum was extracted and applied for ICT strips using standard procedure and protocol. All the ICT positive HCV samples were further processed for viral RNA extraction using Ribo Virus (*Sacace Biotechnologies*, Italy) as manufacturer’s instructions. Active-HCV infection was confirmed through Bio-Rad PCR machine using commercially available InnuPrep®/RoboGene® HCV RNA purification and Quantification kit (Aj-Roboscreen Germany) [15].

### Genomic DNA extraction and T-ARMS PCR for SNP detection

After confirmation of HCV infection, the blood samples were subjected to genomic DNA extraction using standard protocol [16]. ARMS-PCR genotyping method was used to detect the SNP, using specific pairs of primers for *IL-6* rs2069837 and rs17147230 (Table 1). The ARMS PCR reaction was conducted in 10 μL volume containing DNA sample 1 μL, 5 μL of Master mix, 0.5 μL of forward and reverse primer, and 3 μL ddH_2_O. The PCR amplification was done at, initial denaturation 94 °C for 5 min, followed by 35 cycles at 94 °C for 15 s, 57 °C for 15 s and 72 °C for 30 s and final extension at 72 °C for 7 min.

**Table 1:** Specific primers for *IL-6* regions

Region	Primer Name	Sequence (5′–3′)
rs2069837	Outer Forward	CTTCCTGCTGGAACATTCTATGGC
	Outer Reverse	CTTCCTGCTGGAACATTCTATGGC
	Inner Forward	ACTGTGTGCCAGGCACTTTAG
	Inner Reverse	GTTTTGAAGATTAGACACAATATTTATT
rs17147230	Outer Forward	AAAAGGGCAAGGAAGGGAGGTA
	Outer Reverse	CACGAGTCATTTGAGCCATCTTTG
	Inner Forward	CAGCCAATGCTTTGCATGCTT
	Inner Reverse	CAGTGTCATCAGCAGAAACTT

### Gel electrophoresis and data analysis

The ARMS-PCR products were analysed on 2% Agarose gel, comparing with 100 bp DNA Ladder (Thermo Scientific). The odds ratios with corresponding 95% confidence intervals were used to assess association between genetic polymorphism in IL-6 and HCV leading to HCC. A *p*-value < 0.05 was considered statistically significant. Statistical analysis was performed using online MedCalc software. Homozygosis and heterozygosis between the HCC cases and control group were directly counted.

## Results

### Occurrence of HCC in different age groups

**Fig. 1:**
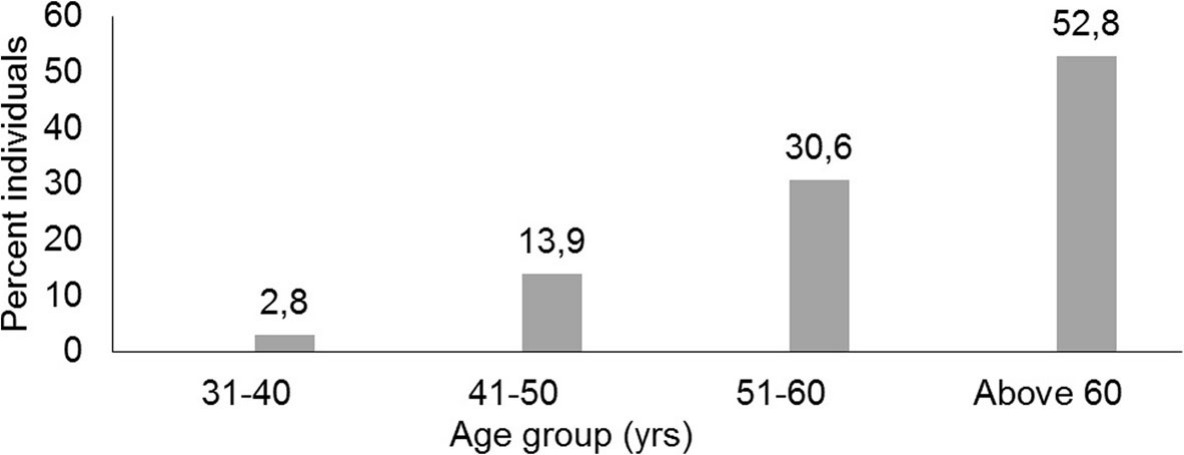
Occurrence of HCC in different age groups

The study was conducted to understand the association of *IL-6* SNPs in HCC patients chronically infected with HCV. Out of 80 HCC and 50 HCV infected patients as control, 72 HCC and 38 HCV were enrolled in this study as our study exclusion/inclusion criteria (HCV leading to HCC). Based on age, patients’ samples were categorized in four different age groups. The age groups of HCC patients were categorized in 31–40 years, 41–50 years, 51–60 years and above 60 years. The occurrence of HCC was noted high in above 60 years (52.8%) followed by 51–60 years (30.6%) (Fig. 1).

### *IL-6* genotyping for region rs2069837 and rs17147230

**Fig. 2:**
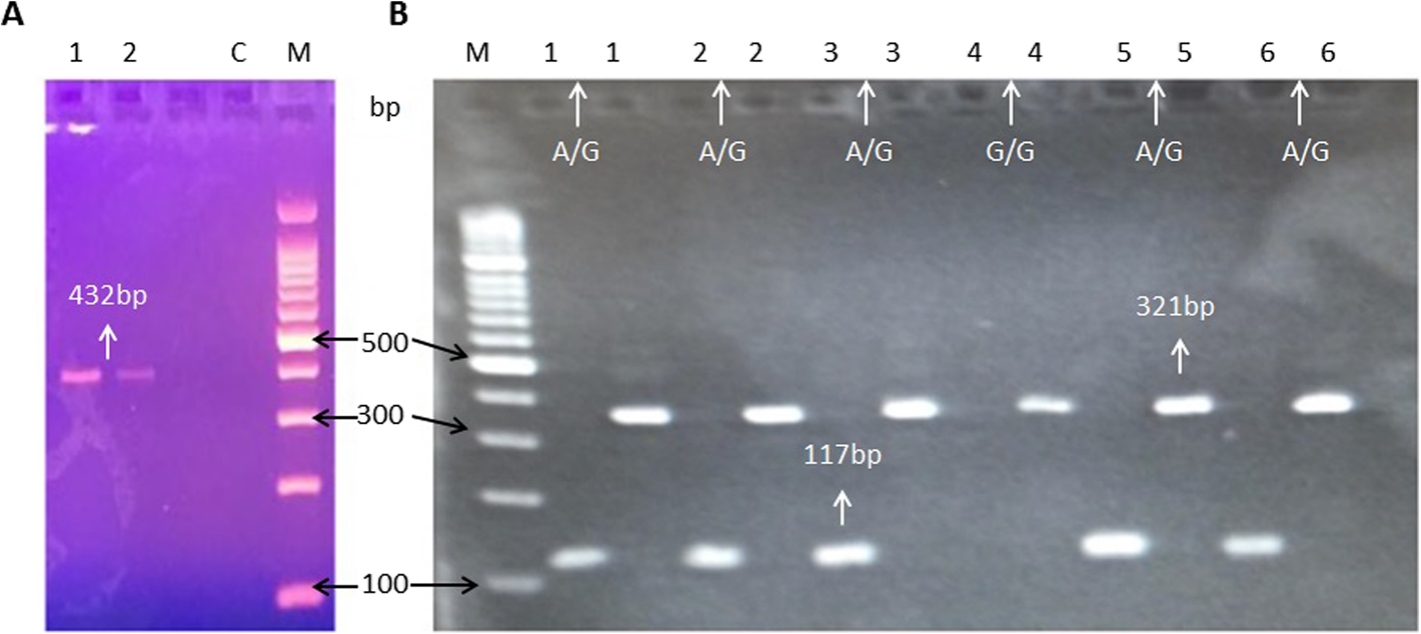
Sample Gel picture of *IL-6* region rs2069837. A region of 432 bp were amplified containing the rs2069837 as control (**a**, lane 1 and 2). For SNP genotyping Forward Outer plus Inner Reverse primers of 117 bp and Inner Forward plus Reverse Outer of 321 bp product sizes were amplified. The AG shows heterozygous mutation (**b**, lane 1,2,3,5 and 6), while GG shows homozygous mutation, lane 4)

**Fig. 3:**
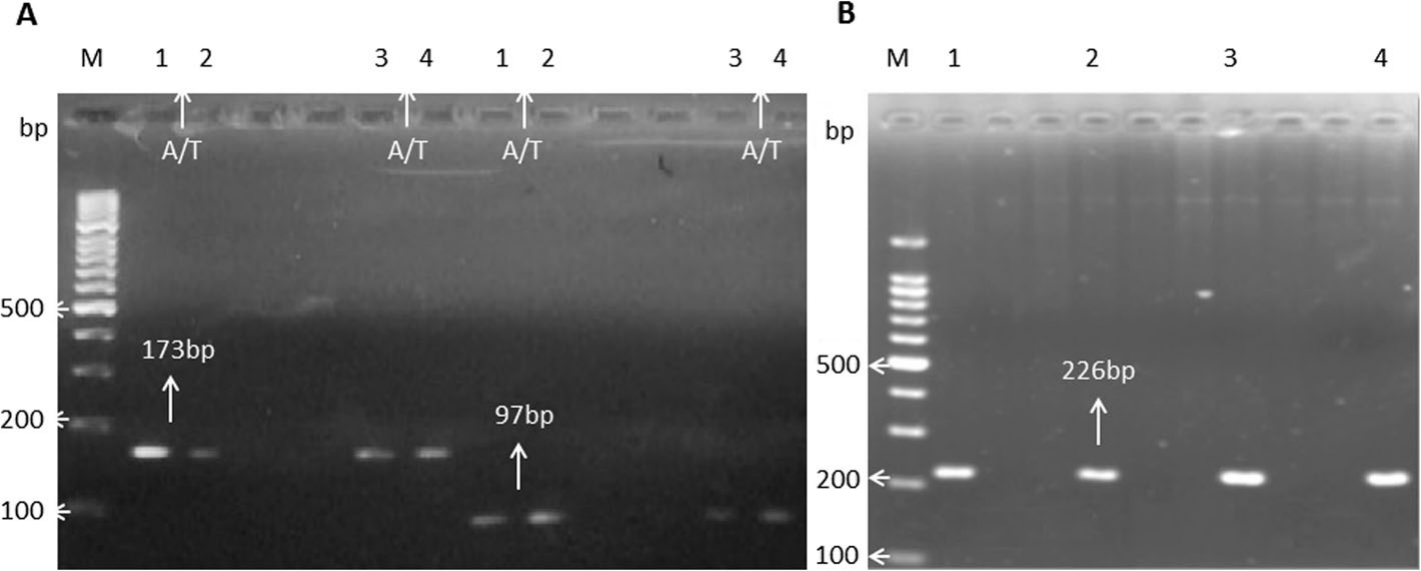
Sample Gel picture of *IL-6* region rs17147230: A region of 226 bp containing the rs17147230 were amplified as control (**b**, lane 1–4). For mutation detection, Forward Outer plus Inner Reverse primers and Inner Forward plus Reverse Outer primers of products sizes 173 bp and 97 bp, respectively, were amplified. The A/T shows heterozygous mutation (**a**, lane 1–4)

All the HCC and control samples were processed for *IL-6* rs2069837 and rs17147230 using T-ARMS PCR. The sample gels pictures for regions rs2069837 and rs17147230, respectively, show the SNPs genotypes (Fig. 2a-b and Fig. 3a-b).

### Frequencies of alleles/genotypes associated with HCC

The molecular analysis shows that the wild A allele of both the regions (rs2069837 and rs17147230) was present in higher frequencies (71.0, 63.0%) in the control group than in the HCV leading to HCC cases (53.0, 53.0%), respectively. Consequently, there was a significant increase in HCC in HCV infected individuals carrying G allele (rs2069837) (OR = 2.196, 95% CI = 1.213–3.978, *p* = 0.0094) and T allele (rs17147230) (OR = 1.432, 95% CI = 0.817–2.509, *p* = 0.210) (Tables 2 and 3). Similarly, the genotype analysis revealed that AA genotype of both the regions was detected in more individuals in control group (63.0%) compared with HCC cases (19.0%). A significant increase in the risk of HCC was noted with the mutations and the heterozygous G allele (rs2069837) (OR = 10.667, 95%CI = 3.923–29.001, *p* = < 0.0001) and T allele (rs17147230) (OR = 75.385, 95%CI = 9.797–580.065, *p* = < 0.0001) carriers were noted to have a significantly higher risk of HCC (Tables 2 and 3). However, under recessive gene model the results were insignificant in case of *IL-6* rs2069837 (OR = 0.605, 95%CI = 0.217–1.689, *p* = 0.337) (Table 2), while significant in case of *IL-6* rs17147230 (OR = 0.298, 95%CI = 0.121–0.734, *p* = 0.0085) (Table 3).

**Table 2:** Statistical relationship and Allele/Genotypes frequencies analysis of rs2069837 Loci in HCC cases and control subjects

Allele/Genotype	Cases	Control	OR	95% CI	*p*-value
	*n = 144*	*n = 76*			
A	76 (0.53)*	54 (0.71)*	Reference		
G	68 (0.47)*	22 (0.29)*	2.196	1.213–3.978	0.0094
	*n = 72*	*n = 38*			
AA	14 (0.19)*	24 (0.63)*	Reference		
AG	48 (0.63)*	6 (0.16)*	10.667	3.923–29.001	< 0.0001
GG	10 (0.14)*	8 (0.21)*	0.605	0.217–1.689	0.337

* Figures in parenthesis indicate allele or genotype frequencies in cases and controls respectively

**Table 3:** Statistical relationship and Allele/Genotypes frequencies analysis of rs17147230 Loci in HCC cases and control subjects

Allele/Genotype	Cases	Control	OR	95% CI	*p*-value
	*n = 144*	*n = 76*			
A	76 (0.53)*	48 (0.63)*	Reference		
T	68 (0.47)*	30 (0.39)*	1.432	0.817–2.509	0.210
	*n = 72*	*n = 38*			
AA	14 (0.19)*	24 (0.63)*	Reference		
AT	48 (0.63)*	0 (0.00)*	75.385	9.797–580.065	< 0.0001
TT	10 (0.14)*	14 (0.37)*	0.298	0.121–0.734	0.0085

* Figures in parenthesis indicate allele or genotype frequencies in cases and controls respectively

## Discussion

Cytokines are important biological molecules that act as soluble mediators of the immune response. IL-6 is produced by numerous typical cells such as mononuclear phagocytes, endothelial cells, T- and B-lymphocytes, fibroblasts, astrocytes and medullary stromal cells. IL-6 induces the synthesis of acute phase proteins, represents the main activator of the differentiation of B lymphocytes into plasma cells and induces the cell cycle in megakaryocytopoietic and myelopoietic progenitors [12].

IL-6 is a pleiotropic cytokine known to play a crucial role in the regulation of the biological effects of hepatocytes and its dysregulated expression has a pathological effect on chronic inflammation, immune response and haematopoiesis.

Chronic inflammation is the result of inflammatory cells recruited to the inflamed site, associated with induction of anti-apoptotic mechanisms [17]. IL-6 plays a key role in this process, due to its dual pro and anti-inflammatory cytokine capacity, which in turn supports cell growth and anti-apoptotic activities that accompany chronic inflammation [18]. Chronic HCV infection is associated with variable outcome, ranging from simple hepatic damage, to cirrhosis and HCC [19]. Furthermore, HCV infection has been shown to play an important role in development of liver disease via the IL-6/STAT3 pathway [20]. Recently, an interesting study has identified a set of immune mediators (cytokines, growth factors, and apoptosis markers) whose levels were significantly higher in serum of patients who eventually developed de novo HCC compared with controls. An higher value of 9 inflammatory cytokines (MIG, IL22, TRAIL, APRIL, VEGF, IL3, TWEAK, SCF, IL21), assumes a possible role in carcinogenesis. These results potentially suggest that before immune changes occur, due to HCV targeting by DAAs, individuals who developed HCC already expressed a differential pattern of immune mediators, possibly induced by ongoing carcinogenic or precarcinogenic activity [21].

Several studies have been conducted to investigate the correlation between the different host genetic factors and the susceptibility of HCV infection in different populations [22–25].

*IL-6* was the most reported gene to be associated with HCV infection or HCC development in chronic HCV patients. The SNPs of the *IL-6* gene have been reported to influence the histologic progression and clinical outcomes of HCV patients [26–28] but there is a great disparity in the correlation between *IL-6* gene polymorphisms and hepatitis-related HCC according to the literature [29, 30].

Polymorphisms of the *IL-6* gene is associated with HCV viral clearance, and the serum IL-6 level shows an increase in HCV infected persons [31] and it has been suggested as biomarker for poor prognosis of patients with HCC [32].

*IL-6* gene plays an important role in human immunologic antagonism, therefore the genetic variations in the *IL-6* gene are commonly studied in HCV infected persons [33]. A few investigations have cited the connection between different ILs gene mutations and incidence of HCV-related HCC progression [28, 34–36]. Thus, the aim of this study is to verify the relationship of *IL-6* gene polymorphism (rs2069837 and rs17147230) with HCV leading to HCC.

Our results indicated that there was a significant HCC increase in HCV infected individuals carrying mutated heterozygous G allele (rs2069837) and T allele (rs17147230) (Tables 2 and 3). However, under recessive gene model the results were insignificant in case of *IL-6* rs2069837, while significant in case of *IL-6* rs17147230. Thus, IL-6 SNPs may be a possible risk factor to contribute to the susceptibility to liver diseases. The genetic polymorphisms of the interleukin genes could influence the infection, pathogenesis and treatment effect of HCV patients [28, 37]. Indeed, the dysregulated synthesis of IL-6 activates downstream immune and oxidative stress signaling to exacerbate inflammation infiltration and eventually leads to the onset or development of liver diseases [30]. Furthermore, elevated IL-6 levels in HCV infected patients are associated with disease progression, probably due to the IL-6 ability to decrease apoptosis of HCC cells, thereby conferring survival advantage for cancer cells [28]. Moreover, IL-6 was linked with natural killer cell dysfunction, which may provide a mechanism of tumor escape from immune surveillance [38].

## Conclusions

HCC pathogenesis is a multistep process involving the progressive accumulation of genetic and epigenetic alterations. Molecular pathogenesis is extremely complex and heterogeneous and this reflex the lack of specific molecular characterization. Thus, direct molecular or epigenetic research is needed to investigate the actual scenario of HCC in the context of HCV viral infection, that leading liver to steatosis, cirrhosis, or eventually HCC.

In our study we observed a significant association for both the SNPs within *IL-6* with HCC susceptibility. More intensive investigations will be required to explore the possible involvement of *IL-6* polymorphisms to HCC progression in HCV infected individuals, with the aim of future application of SNPs as biomarkers for the risk stratification of HCC onset and the prediction of the prognosis, as well as to evaluate the clinical progress of the disease.

## Data Availability

All data generated or analysed during this study are included in this published article.

## Abbreviations

CI: Confidence interval
DAAs: Direct-acting antivirals
ddH_2_O: Double-distilled water
DNA: Deoxyribonucleic acid
GCP: Good clinical practice
HBV: Hepatitis B virus
HCV: Hepatitis C Virus
IBGE: Institute of Biotechnology and Genetic Engineering
ICH: The International Conference on Harmonisation of Technical Requirements for Registration of Pharmaceuticals for Human Use
ICT: Immunochromatographic test
IL-6: Interleukin-6
KP: Khyber Pakhtunkhwa
OD: Odds ratio
SNPs: Single nucleotide polymorphisms
STAT3: Signal transducer and activator of transcription 3 protein
T-ARMS-PCR: Tetra-primer amplification refractory mutation system based polymerase chain reaction
UAP: University of Agriculture Peshawar
